# Synergistic Effect of Combined Walnut Peptide and Ginseng Extracts on Memory Improvement in C57BL/6 Mice and Potential Mechanism Exploration

**DOI:** 10.3390/foods12122329

**Published:** 2023-06-09

**Authors:** Junxi Fu, Wentian Song, Xiaobing Song, Li Fang, Xiyan Wang, Yue Leng, Ji Wang, Chunlei Liu, Weihong Min

**Affiliations:** 1 College of Food Science and Engineering, Jilin Agricultural University, Changchun 130118, China; fujunxi0401@163.com (J.F.); songwentiana@163.com (W.S.); fangli1014@126.com (L.F.); wangxiyan199294@163.com (X.W.); lengyue@jlau.edu.cn (Y.L.); wangji198644@163.com (J.W.); 2 National Engineering Laboratory of Wheat and Corn Deep Processing, Changchun 130118, China; 3 Zhongke Special Food Institute, Changchun 130022, China; songguo1008@163.com

**Keywords:** walnut peptide, memory improvement, ginseng extracts, neurotransmitter, synergistic effect, hippocampus

## Abstract

This work aimed to investigate whether there are synergistic effects between walnut peptide (WNP) and ginseng extracts (GSE) treatments to ameliorate the memory impairment caused by scopolamine (SCOP). The Morris water maze trial, hippocampal neuron morphology, neurotransmitters, and synaptic ultrastructure were examined, along with brain-derived neurotrophic factor (BDNF)-related signaling pathway proteins. The results of the Morris water maze trial demonstrated that the combined administration of WNP and GSE effectively alleviated memory impairment in C57BL/6 rats caused by SCOP. Improvement in the morphology of hippocampal neurons, dendritic spines, and synaptic plasticity and upregulation of neurotransmitters AChE, ACh, ChAT, Glu, DA, and 5-HT supported the memory improvement effects of WNP + GSE. In addition, compared with the model group, WNP + GSE significantly enhanced the protein levels of VAChT, Trx-1, and the CREB/BDNF/TrkB pathway in hippocampal and PC12 cells induced by SCOP (*p* < 0.05). Notably, WNP + GSE boosted memory via multiple pathways, not only the BDNF/TrkB/CREB target.

## 1. Introduction

A global increase in the number of aging people has led to a surging incidence of neurodegenerative diseases, such as Alzheimer’s disease (AD) and vascular dementia, mainly characterized by the decline or loss of learning and memory capacity [1]. Among individuals over 65 years of age, the risk of developing AD has grown to 10%, and ~150 million adults are predicted to suffer from AD by 2050 [2]. The onset of AD typically involves initial memory impairment, which is then followed by a range of complex functional disorders, including visual–spatial disorders, personality disorders, and executive dysfunction. Timely intervention in cognitive impairment plays a critical role in delaying the progression of the disease [3]. However, according to the multifactorial nature of AD, single substances can rarely lead to definitive management. Natural products and foods could be investigated more as novel interventions for AD and also as valuable sources of bioactive compounds. Multitarget approaches via natural medications in traditional systems of medicine could be reconsidered for more effective therapies [4].

Walnut (*Juglans mandshurica Maxim*.) is an important tree nut, and walnuts have been extensively studied as preventive agents for brain atrophy and memory loss and are considered an effective nutrient for preventing and managing Alzheimer’s disease [5]. In studies conducted by Persian medical scholars, the therapeutic benefits of walnuts were documented through clinical observations, supported by extensive historical evidence. Notably, peptides derived from walnuts have gained recognition for their favorable absorption characteristics and their ability to modulate cognitive function. Studies have shown that walnut protein hydrolysates play a protective role in scopolamine-induced learning and memory deficits, D-galactose and aluminum chloride-induced neurotoxicity, and Aβ_25–35_-induced memory impairment [6]. Our previous study demonstrated that the three peptides TWLPLPR, YVLLPSPK, and KVPPLLY derived from walnut (*Juglans mandshurica Maxim*.) hydrolysate with molecular weights (MW) < 3 KDa can improve cognitive impairment [7]. Ginseng (*Panax ginseng Meyer*) is a traditional Chinese medicinal plant sharing cognitive-enhancing properties [8]. Ginseng extract (GSE) contains important bioactive compounds and components. Ju et al. [9] demonstrated that GSE improved learning and memory capacities in mice by increasing antioxidant enzymes in the hippocampus via upregulation of Nrf2-mediated antioxidant mechanisms. Zhu et al. [10] showed that GSE reduced apoptosis through upregulation of Bcl2 and downregulation of Bax levels and attenuated cognitive dysfunction in rats with vascular dementia. It is worth noting that the biological activities of natural components can be influenced by their interactions with one another. Wu et al. [11] confirmed that combination of the peptides QMDDQ and AGLPM isolated from shrimp and maize improved cognition better than either peptide alone. Sheng et al. [12] found that the combination of tea polyphenols and proanthocyanidins attenuated cognitive impairment and reversed neurodegenerative lesions, which were induced by Aβ_1–42_ in mice. However, limited studies have reported the combined effect of bioactive peptides and ginseng extracts in enhancing memory capacity, and whether the peptides and GSE work synergistically on improving cognitive impairment is as yet unknown.

While the precise mechanism underlying the pathogenesis of AD remains largely unclear, the regulation of neurotransmitters and the cholinergic system plays a vital role in enhancing cognitive function in neurodegenerative disorders. Beyond their role in transmitting and amplifying neural signals between nerves and other cell types, neurotransmitters also serve as key regulators of neuronal growth and differentiation [13]. Neurotransmitters communicate between presynaptic and postsynaptic neurons and thus maintain synaptic and cognitive functions in mammals. Therefore, synapses exhibit multiple forms of short-term plasticity, mostly attributed to neurotransmitter release. Notably, synaptic plasticity is important in transforming transient memory into permanent memory and is closely associated with long-term potentiation (LTP) and long-term depression [14]. Early symptoms of cognitive impairment include neurotransmitter loss, impaired cholinergic function, and disruption of synaptic plasticity, which eventually weakens electrical and chemical signals of neurons and damage synapses [15].

In this work, we aimed to determine if there is a synergistic interaction between Juglans mandshurica Maxim-derived walnut peptides (WNP) and ginseng extracts (GSE) on memory-improving activity. To assess the impact of WNP + GSE on learning and memory functions in mice with SCOP-induced cognitive impairment, various techniques, including the Morris water maze trial, Nissl staining, HE staining, Golgi staining, and ultrastructure observations, were utilized. Further, neurotransmitter assays were conducted and protein levels of the CREB/BDNF/TrkB pathway in both hippocampal neurons and PC12 cells with the treatment of activators and inhibitors were determined to explore possible mechanisms of WNP + GSE in enhancing learning and memory ability.

## 2. Materials and Methods

### 2.1. Materials and Reagents

Five-year-old dried white ginseng was purchased from Lusongtang Ginseng Co., Ltd. (Baishan, China). Walnut peptide (WNP) was prepared according to Ren et al. [16]. GSE was prepared using the following conditions: a material: liquid ratio of 1:10, sonication at 75 °C for 47 min, and water decoction at 100 °C for 2.5 h. WNP + GSE is a combination of walnut peptide (WNP) and lyophilized ginseng extracts (GSE), with WNP comprising 30% and GSE comprising 70% of the mixture, respectively. Assay kits for acetylcholine (ACh), acetylcholinesterase (AChE), dopamine (DA), 5-hydroxytryptamine (5-HT), choline acetyltransferase (ChAT), glutamate (Glu), cAMP response element-binding protein (CREB), phosphorylated cAMP response element-binding protein (p-CREB Ser133), BDNF, tyrosine kinase B (TrkB), phosphorylated tyrosine kinase B (p-TrkB Y705), thioredoxin 1 (Trx-1), vesicular acetylcholine transporter protein (VAChT), and β-actin were purchased from Abcam (Cambridge, MA, UK). The inhibitor ANA-12 (a TrkB receptor system blocker) and activator 7,8-dihydroxyflavone (7,8-DHF) were purchased from APExBIO (APExBIO Technology LLC, Houston, TX, USA), and all secondary antibodies were purchased from ABclonal Co. Ltd. (Wuhan, China).

### 2.2. Animal Quarantine and Procedure

Animal experiments were approved by the Ethics Committee of Jilin Agricultural University and were carried out following the *Guide for the Care and Use of Laboratory Animals* published by the European Commission. A total of fifty male C57BL/6 mice aged 5–6 weeks and weighing 20 ± 2 g were obtained from Liaoning Changsheng Biotechnology Co., Ltd. (Shenyang, China) for the purpose of this study. The mice were housed at the Experimental Animal Center of Jilin Agricultural University, where they were maintained under controlled conditions, including a stable temperature of 22 ± 1 °C, relative humidity of 55 ± 5%, and a 12 h light/dark cycle. They were provided with ad libitum access to standard chow and water. After acclimatization of one week, mice were randomized into 5 groups (10 mice/group): (I) 0.9% saline gavage for 37 days (control group); (II) 0.9% saline gavage for 30 days followed by intraperitoneal (i.p.) injection of SCOP (2 mg/kg) for 7 days (model group); (III) 0.9% saline gavage for 30 days followed by i.p. injection of piracetam (60 mg/kg) + SCOP (2 mg/kg) for 7 days (positive group); (IV) WNP gavage for 30 days (600 mg/kg) followed by WNP + SCOP (2 mg/kg) for 7 days (WNP group); and (V) WNP + GSE gavage for 30 days (600 mg/kg) followed by the combination of WNP and GSE + i.p. injection of SCOP (2 mg/kg) for 7 days (WNP + GSE group).

### 2.3. Morris Water Maze Trial

The Morris water maze trial was performed following the method of Zhao et al. [7] with slight modifications and included a training phase and a final test phase. During the training phase, a platform was positioned and secured 1 cm below the water surface in the southwest quadrant of a pool. For 7 consecutive days, the mice were carefully introduced into the water facing the wall of the pool. If a mouse successfully located the platform within 120 s, it was allowed to remain on the platform for 10 s. If a mouse did not find the platform within the time limit, it was gently guided to the platform using a wooden stick and allowed to stay there for 10 s. In the final test phase, the mice were placed in the water without any assistance or guidance to locate the platform. A computer was used to record their latency, total movement distance, and movement time and distance in effective zones. For the spatial exploration test, the platform used for the Morris water maze (MWM) test was removed. Then, the mice were put into the water from the northeast quadrant. A computer recorded the number of times the mice entered the escape platform within 90 s, the residence time on the escape platform, effective movement distance, and effective movement time. The above tests were performed 30 min following drug injection.

### 2.4. Determination of Biochemical Indexes

Neurotransmitters and oxidative stress indicators were measured in mouse serum. After the behavioral tests, blood was taken from the eyeball and naturally clotted at room temperature for 20 min. Supernatant was collected following centrifugation at 3500× *g* for 20 min at 4 °C. AChE, ACh, ChAT, Glu, DA, 5-HT, GSH-Px, SOD, and T-AOC levels were measured following the instructions of the corresponding kits.

### 2.5. Brain Morphological Changes

Hematoxylin and eosin (HE) staining: The mice were killed. Their brains, livers, and kidneys were extracted and fixed with 4% polymethanol followed by dewaxing in xylene for 10 min, dehydration using 70–100% gradient ethanol, rinsing with distilled water for 5 min, staining with hematoxylin for 5 min, rinsing with distilled water for 1 min, destaining with 1% hydrochloric acid for several seconds, and final staining with eosin for 10 min.

Nissl staining: Slides were stained with 0.5% toluene violet solution for 15 min, dehydrated with ethanol gradients (70%, 95%, and 100%), placed in xylene, and then covered with mounting medium. Subsequently, the morphological and structural alterations in hippocampal, liver, and renal cells of mice were examined using a light microscope, and corresponding images were captured (Nikon DS-U3, Tokyo, Japan).

### 2.6. Immunohistochemistry

Mouse hippocampal tissue sections were placed in an antigen repair kit, boiled over medium heat for 8 min, and then heated over low heat for 7 min. Following natural cooling, the samples underwent three washes in PBS (pH 7.4) for 5 min each. Subsequently, they were incubated with 3% hydrogen peroxide in the dark at room temperature for 25 min, followed by three additional washes. The samples were then blocked with serum for 30 min. Next, they were incubated with BDNF and VAChT antibodies at 4 °C, followed by three washes. Finally, the samples were incubated with the secondary antibody for 50 min at room temperature. After washing thrice, freshly prepared 2,4-diaminobutyric acid (DAB) chromogenic solution was added to the sections and then observed under the microscope (Nikon E100, Tokyo, Japan) for image acquisition and analysis (Nikon DS-U3, Tokyo, Japan).

### 2.7. Golgi Staining

After brain tissue samples had been fixed for 48 h, they were sectioned into 2–3 mm-thick tissue blocks. The sections underwent a gentle rinsing with 0.9% saline solution, followed by immersion staining for 48 h. This staining process involved submerging the sections in Golgi staining solution contained in 45 mL round-bottomed EP tubes. To ensure optimal staining, the dye was replaced with fresh solution every 3 days over a period of 14 days. The entire procedure was carried out in a dark environment. Subsequently, the tissues were washed three times with distilled water and then immersed overnight in 80% glacial acetic acid. After becoming soft, the tissues were washed with distilled water, placed in 30% sucrose solution, sectioned into 100 µm thickness, and then dried overnight on gelatin slides. These sections were treated with concentrated ammonia and acidic firm film fixative for 15 min, then dried and sealed with glycerol gelatin. They were imaged (400×) using CaseViewer 2.4 scanning software. Image-Pro Plus 6.0 analysis software (Media Cybernetics, Inc., Rockville, MD, USA) was used to quantify the dendritic spines present in a 30–90 µm-length range of the 2nd or 3rd dendritic branch of the intact central neuron along with measuring their length.

### 2.8. Transmission Electron Microscope (TEM) Observation

As reported earlier, the CA1 region of the hippocampus was dissected and sliced into 1 mm sections. Next, the sections were rinsed three times (10 min each) with 0.1% phosphate buffer solution at pH 7.4. Subsequently, the sections were fixed in the dark using 1% osmium acid for 2 h at room temperature. After fixation, the sections underwent dehydration through an ethanol gradient following several washes. This was followed by embedding, baking in an oven at 60 °C for 48 h, and staining. Neurons and synapses were imaged with a TEM (H-7650, Hitachi, Tokyo, Japan). Different fields of the 700 µm ultrathin sections were randomly selected for imaging. The thickness, length, posterior chord length, arc length, and gap in the postsynaptic density (PSD) were measured using Image-Pro Plus 6.0 software.

### 2.9. Western Blot

Phenylmethylsulfonyl fluoride (PMSF) containing proteolytic enzymes (90 µL) was added per mg of hippocampal tissue. Then, the tissues were ground at low temperature, shaken for 30 min, and centrifuged at 12,000× *g* for 10 min at 4 °C. The supernatant containing protein was collected and stored at −20 °C till further analysis. Protein estimation was performed using a bicinchoninic acid (BCA) kit. To separate the proteins, sodium dodecyl sulfate (SDS)–polyacrylamide gel electrophoresis (PAGE) was performed. The separated proteins were subsequently transferred onto polyvinylidene fluoride (PVDF) membranes using a constant current of 200 mA/cm^2^ for 30 min. Following this, the membranes were blocked in 5% skim milk solution in Tris-buffered saline–Tween (TBST) for 1 h and then washed three times with TBST for 5 min each. They were further incubated separately with the primary antibodies BDNF (ab108319, 1:1000), TrkB (ab187041, 1:1000), CREB (4619, 1:1000), p-CREB (9198, 1:1000), VAChT (ab235201, 1:1000), and β-actin (8457, 1:1000) at 4 °C overnight, followed by another incubation with horseradish peroxidase (HRP)-labeled secondary antibody (1:1000) at 24 °C for 1 h. Lastly, the chemiluminescent substrate (Bio-Rad Laboratories, Inc., Hercules, CA, USA) was applied uniformly on the membranes and was developed 2 min post-incubation. The Western blots were analyzed using ImageJ software (version 2.0.0).

### 2.10. Cell Cultures

PC12 cells were cultured in RPMI 1640 medium containing 10% fetal bovine serum at 37 °C with 5% CO_2_. The cells were seeded on 6-well plates (2 × 10^5^ cells per well) for 24 h, cultured with components WNP and WNP + GSE (400 µM) for 24 h, and treated with 4 mg SCOP for 4 h. The cells in the control group were not treated, and those in the model group were treated with SCOP only. In the inhibitor group, the cells were cultured with a concentration of 10 µM ANA-12 for 1 h. Conversely, the cells in the activator group were incubated with 25 µM 7,8-DHF for 1 h. Cell viability assays were conducted using 3-(4,5-dimethylthiazol-2-yl)-2,5-diphenyltetrazolium bromide (MTT) obtained from Sigma-Aldrich, St. Louis, MO, USA.

### 2.11. Statistical Analysis

Statistical analysis and figure generation were performed using GraphPad Prism software (version 8.3.1). All data are presented as means ± standard deviation (SD). Results were analyzed using one-way ANOVA, followed by Tukey’s test. *p*-values < 0.05 indicate statistical significance.

## 3. Results and Discussion

### 3.1. Effects of WNP + GSE on Learning and Memory Functions in Mice with SCOP-Induced Cognitive Impairment

**Figure 1:**
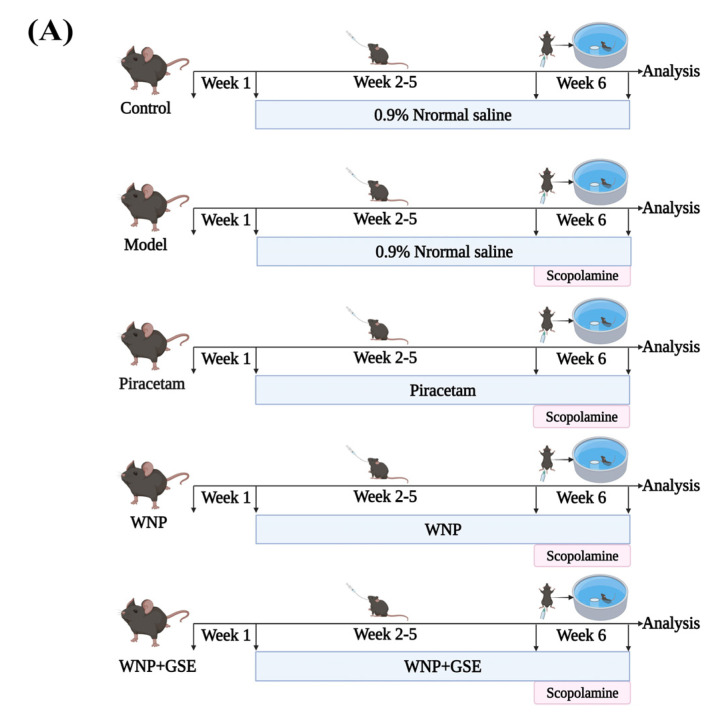

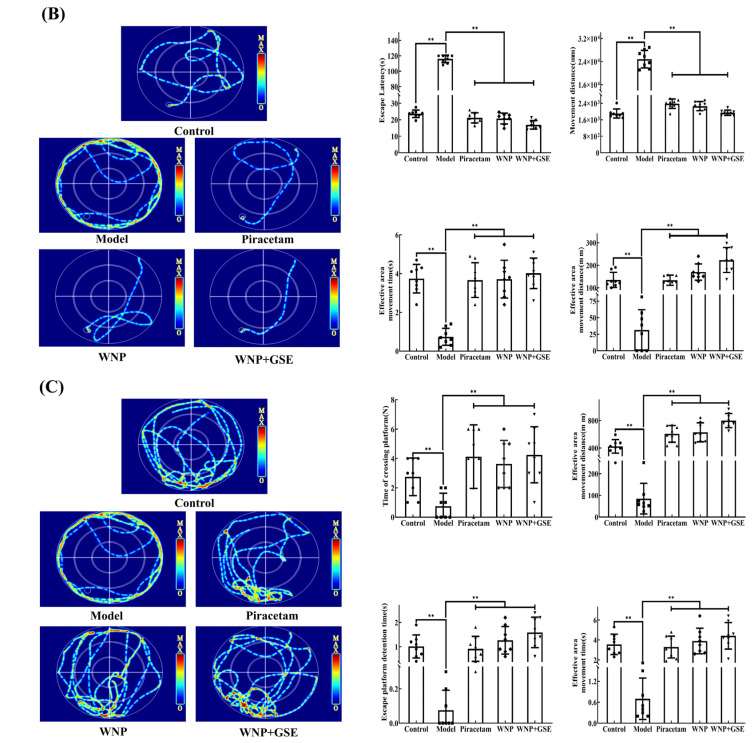
Effects of WNP + GSE on learning and memory deficits of SCOP–induced mice (**A**) Flow diagram of experimental tests. (**B**) Representative image of movement trajectory of mice in the positioning navigation test, including escape latency, total movement distance, effective movement time, and effective movement distance. (**C**) Representative image of movement trajectory of mice in the spatial exploration test, including the number of times mice crossed the original platform, effective movement distance, escape residence, and effective movement time. N = 8 mice/group. Data are expressed as means ± standard deviation. Asterisks indicated a significant difference, ** *p* < 0.01. All experiments were repeated three times.

The experimental protocol used to determine the effect of WNP + GSE on SCOP-induced cognitive impairment in mice is shown in Figure 1A. The cognitive function of the mice was evaluated through the MWM test. The model mice, induced by SCOP, exhibited erratic and aimless movement patterns in the spatial navigation test, in contrast to the control mice. However, the mice in the WNP + GSE group displayed faster discovery of the hidden platform compared to the model mice, indicating an improved memory capability (Figure 1B). The MWM test showed that the escape latency of the WNP + GSE group (16.93 ± 2.43 s) was lower than that of the model group (116 ± 4.58 s; *p <* 0.05). In addition, the total distance covered by the WNP (2261.85 ± 208.69 mm) and WNP + GSE group (1942.98 ± 118.37 mm) was significantly lower than the model group (24,858.56 ± 2831.02 mm; *p <* 0.05). Notably, the effective area movement time and distance covered by the WNP + GSE group mice (4.01 ± 0.74 s and 223.14 ± 51.96 mm) were significantly higher than those in the model group (0.74 ± 0.41 s and 31.44 ± 28.50 mm; *p <* 0.05). In the spatial exploration test, the initial submerged platform was removed, and the frequency of the mice’s search for the original platform within a specific time frame was utilized as an indicator of cognitive function. The findings revealed that mice in the WNP + GSE group exhibited a significant preference for the target quadrant when compared to the mice in the model group (Figure 1C). The number of crosses over the original platform was higher in the WNP + GSE group (4.25 ± 1.79 N) than in the model group (0.75 ± 0.83 N; *p <* 0.05). Additionally, the effective movement area distance covered by the WNP + GSE group (798.6 ± 96.78 mm) was significantly higher than the model group (84.84 ± 66.24 mm), and the escape detention time (1.59 ± 0.59 s) and effective area movement time (4.41 ± 1.24 s) in the WNP + GSE group were significantly higher than the model group (0.075 ± 0.11 s and 0.7 ± 0.55 s, respectively) (*p <* 0.05).

The MWM test is one of the most extensively utilized and authoritative approaches for evaluating spatial learning and memory functions dependent on the hippocampus in rodents. Numerous studies employing the MWM test have demonstrated that exposure to SCOP can negatively affect the learning and memory capabilities of mice. Piracetam, the prototype of the so-called nootropic drugs, has been used for many years in different countries to treat cognitive impairment in aging and dementia. Substantial evidence for elevated neuronal plasticity as a specific effect of piracetam has emerged [17,18], so it was used as a positive control in this work. Wu et al. [19] showed that a SCOP-induced mice model group had deficiencies in cognitive and memory abilities and longer escape latency compared to the control group, confirming that intraperitoneal injection of SCOP causes cognitive impairment. Zhao et al. [7] discovered that SCOP-induced mice treated with the walnut-derived peptide YVLLPSPK had significantly reduced latency, increased number of entries into the escape platform, and longer escape residence time. Further, Shi et al. [20] reported that the combination of ginsenoside Rg1 and *Acori graminei* rhizome significantly shortened the mean number of crossings and residence time in the target quadrant by SAMP8 mice and improved the viability of SAMP8-injured PC12 cells. The findings of our experiment revealed that the administration of WNP + GSE in SCOP-induced mice led to an improvement in cognitive impairment.

### 3.2. Regulation of Hippocampal Neuron Morphology by WNP + GSE

**Figure 2:**
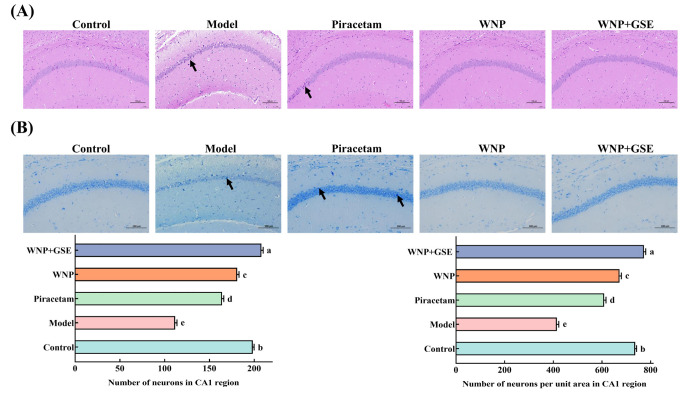

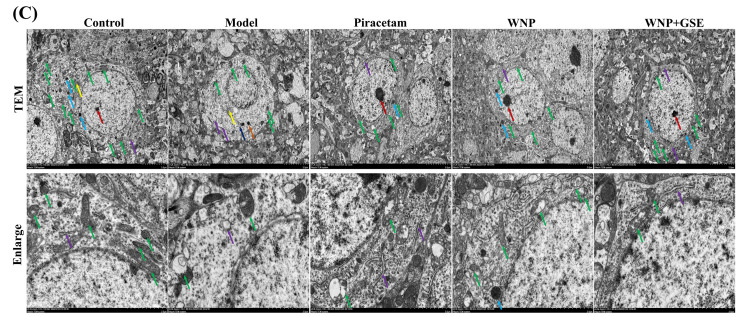
Effect of WNP + GSE on the morphology of mouse hippocampal neurons and dendritic spine density. (**A**) HE pathological sections of mouse hippocampus: black arrows indicate deep staining and pyknosis. Scale bars = 100 µm. (**B**) Nissl staining of the number of neurons and neurons per unit area in the mouse hippocampus: black arrows indicate deep staining and pyknosis. Scale bars = 100 µm. (**C**) TEM image of neuronal pathology. Scale bars = 5 µm. Data are expressed as means ± standard deviation; all experiments were repeated three times; N = 3. Different letters represent significant differences, *p* < 0.05.

Hippocampal neuron morphology was observed by HE staining, Nissl staining and high-resolution TEM. The hippocampal neurons of control mice were neatly and tightly organized without cell number reduction using HE staining, and the cell structure was intact and clear, with no pyknosis and a distinct nucleoplasmic boundary. On the other hand, the neurons in model mice were loosely arranged and disordered, had enlarged gaps, and had degenerated cells with nuclear pyknosis and blurred nucleoplasmic boundaries. Compared to the WNP-treated group, the neurons in the WNP + GSE group were more normal in structure and arranged more closely and neatly, with clear nucleoplasmic boundaries and no significant degeneration (Figure 2A). Additionally, Nissl staining revealed distinct morphological differences between the neurons in the model group and the control group. Neurons in the model group displayed a less organized arrangement, indistinct cell outlines, absence of cytoplasmic Nissl bodies, lighter staining, signs of cellular degeneration, cytosolic consolidation, and more intense staining compared to the control group. In contrast, neurons in the WNP + GSE group exhibited a more orderly and compact arrangement, clearer nuclei, and a higher abundance of cytoplasmic Nissl bodies when compared to the WNP group. The number of neurons and the number of neurons per unit area of the CA1 region were counted. It was found that the number of neurons in the hippocampal CA1 region was higher in the WNP + GSE group (208 ± 1.63) compared to the WNP group (181 ± 1.63) (*p <* 0.05). In addition, the number of neurons per unit area was significantly higher in the WNP + GSE group (772.38 ± 6.06) than in the WNP group (672.12 ± 6.06) (Figure 2B; *p <* 0.05). The number of neurons and the number of neurons per unit area increased after WNP + GSE treatment compared to the control group (198.33 ± 1.25 and 736.49 ± 4.63; *p <* 0.05).

TEM analysis of pyramidal neurons in the CA1 region of the mouse hippocampus showed notable structural alterations in the model group. These alterations included moderate edema, a sparse intracellular matrix, organelle swellings, blurred and locally lysed nuclear membrane, and a slightly widened perinuclear gap. Moreover, the mitochondria (indicated by green arrows) exhibited swelling and partial enlargement, along with the presence of broken and reduced cristae, as well as a decreased electron density of the intramembrane matrix. The WNP group neurons had mild edema, uniform intracellular matrix electron density, and mild swelling of organelles compared to the model group. Their nuclei had a subcircular shape, clear double nuclear membrane structure, no obvious widening of the perinuclear gap, and uniform chromatin. The numbers of mitochondria (shown by green arrows) were more, with their cristae mostly broken and reduced and reduced electron density of the intramembrane matrix. Compared with the WNP group, the neurons in the WNP + GSE group were slightly swollen, with obvious nuclei, centered, and abundant in mitochondria (shown by green arrows) (Figure 2C).

The hippocampal CA1 region plays a crucial role in discrimination and spatial processing. Memory impairment often coincides with the presence of lesions and neuronal death in the hippocampus, resulting from morphological damage. These detrimental effects ultimately contribute to a decline in learning and memory capacity [21]. Recent studies have suggested that promoting neuronal integrity can boost cognitive function and memory. Chen et al. [22] found that ginsenoside Rb1 can increase neuronal numbers and treat cognitive impairment caused by cisplatin. Zhao et al. [23] showed that sea cucumber-derived peptide FYDWPK can repair neuronal morphology and increase the number of Nissl bodies to reverse SCOP-induced memory impairment in mice. Consistent with the above findings, WNP + GSE treatment improved the morphology and number of hippocampal neurons in mice.

### 3.3. WNP + GSE Improved Synaptic Plasticity in the Mouse Hippocampus

**Figure 3:**
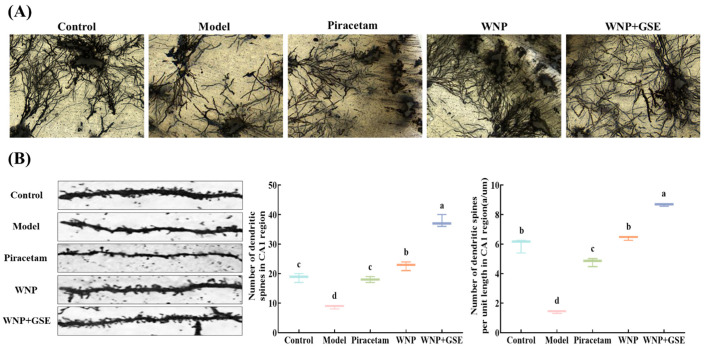
Golgi staining image of mouse hippocampus. (**A**) Golgi staining image of the hippocampal CA1 region. (**B**) Representative dendritic spine localization in the hippocampal CA1 region; the number of dendritic spines in the hippocampal CA1 region, and the number of dendritic spines per unit length. Scale bars = 100 µm. Data are expressed as means ± standard deviation; all experiments were repeated three times; N = 3. Different letters represent significant differences, *p* < 0.05.

It has been reported that in clinical patients or animal models, structural degeneration, such as reduction in neurons, generally does not appear until the middle-late stage, and cognitive impairment in the early stage of the disease is more likely to be caused by abnormal synaptic function in specific brain regions (prefrontal cortex and hippocampus) [24]. In the brain, excitatory glutamatergic synapses are often made on dendritic spines that enlarge during learning. Dendritic spines and the presynaptic terminals are tightly connected with the synaptic cleft; the enlargement may have mechanical effects on presynaptic functions [25]. Therefore, dendritic spines and synaptic plasticity have long been considered important components of learning and memory [26]. Analysis of Golgi staining revealed that the density of dendritic spines in the hippocampal neurons of mice in the model group was low. However, after the WNP + GSE treatment, the density of dendritic spines increased, as shown in Figure 3A. Dendritic spine images were captured for each group, as depicted in Figure 3B, and the number of dendritic spines as well as the density of dendritic spines per unit length were quantified. The number of dendritic spines was significantly increased in the WNP + GSE group (37.67 ± 1.70) compared to the WNP group (22.67 ± 1.25, *p <* 0.05), and the number of dendritic spines per unit length was considerably higher in the WNP + GSE group (8.66 ± 0.06) compared to the WNP group (6.41 ± 0.10) (*p <* 0.05). Meanwhile, the number of dendritic spines and dendritic spines per unit length was higher in the WNP + GSE group than in the control group (18.67 ± 1.25 vs. 1.41 ± 0.08; *p <* 0.05).

**Figure 4:**
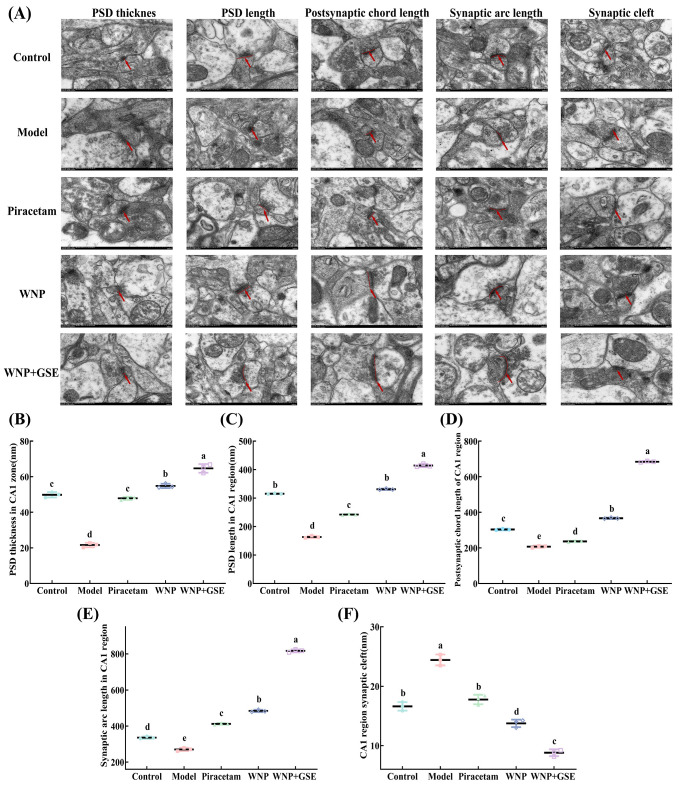
TEM observation of synaptic ultrastructure in the mouse hippocampus. (**A**) Imaging of synaptic ultrastructure. (**B**) PSD thickness in the hippocampal CA1 region. (**C**) PSD length in the hippocampal CA1 region. (**D**) Postsynaptic chord length in the hippocampal CA1 region. (**E**) Arc length in the hippocampal CA1 region. (**F**) Synaptic gap in the hippocampal CA1 region. Data are expressed as means ± standard deviation; all experiments were repeated three times; N = 3. Different letters represent significant differences, *p* < 0.05.

The ultrastructure of synapses in the hippocampal region was observed by TEM (Figure 4A). The thickness and length of the PSD were significantly increased in the WNP + GSE group (71.34 ± 1.36; 414.31 ± 4.70) compared to the WNP group (54.83 ± 0.99; 330.95 ± 1.92) (Figure 4B,C). Moreover, the length of the postsynaptic chord and arc exhibited a significant increase in the WNP + GSE group (684.20 ± 3.08 and 816.56 ± 6.60, respectively) compared to the WNP group (366.64 ± 2.91 and 484.04 ± 5.36, respectively; *p <* 0.05) Figure 3E and Figure 4D). Synaptic vesicles play a crucial role in the transportation of neurotransmitters, which are subsequently released into the synaptic cleft. A small synaptic gap attenuates the delay in transmitting signals; therefore, the size of the synaptic gap also affects synaptic plasticity and cognitive performance. The results showed that the synaptic gap in the WNP + GSE group (8.79 ± 0.47) was significantly lower than that in the WNP group (13.74 ± 0.52) and control group (16.61 ± 0.59) (*p <* 0.05).

The morphogenesis and plasticity of dendritic spines are associated with synaptic strength, learning, and memory. Li et al. [27] demonstrated that treatment with ginsenoside Rg1 significantly improved dendritic spines, enhanced neuronal resistance to apoptosis, and resisted oxidative stress attacks on the brain. Wang et al. [28] reported that ginsenoside Rb1 restored neuronal numbers and improved dendritic spine density and synaptic plasticity. The density of the dendritic spine is related to the formation of excitatory synapses and affects the establishment of proper neuron connectivity [29]. The findings of this study demonstrate that the combined treatment of WNP and GSE exerted the most pronounced improvement in dendritic spine density.

On the other hand, changes in the synaptic structure are important for the expression and storage of information in neuronal networks and may contribute to the formation of long-term memory, making synaptic plasticity a major mechanism associated with learning and memory [30]. WYPGK peptide, isolated from pine nut protein hydrolysate, improved synaptic plasticity by enhancing the thickness of PSD and the arc length of the postsynaptic membrane, resulting in the reversal of SCOP-induced cognitive impairment [31]. Donovan et al. [32] found that ginsenoside Rb1 improved synaptic plasticity by elevating synaptic marker protein content and protected mice from MPTP-induced cognitive impairment. Wang et al. [28] reported that the ginsenoside Rb1 significantly attenuated synaptic gaps and enhanced synaptic functional transmission. These findings demonstrate that the combined presence of multiple ginsenosides in WNP + GSE exhibits a synergistic effect, leading to a more effective improvement in the thickness, length, postsynaptic chord length, and arc length in the PSD compared to WNP alone. Consequently, this enhances synaptic plasticity, rescues cognitive impairment induced by SCOP, reduces synaptic gaps, and improves signal transmission. Neurotransmitters are transmitted via synapses between neurons, and their release promotes neuronal development and regulates synaptic plasticity. As such, we simultaneously measured the effects of WNP + GSE on neurotransmitter release.

### 3.4. WNP + GSE Promotes the Release of Neurotransmitters

**Figure 5:**
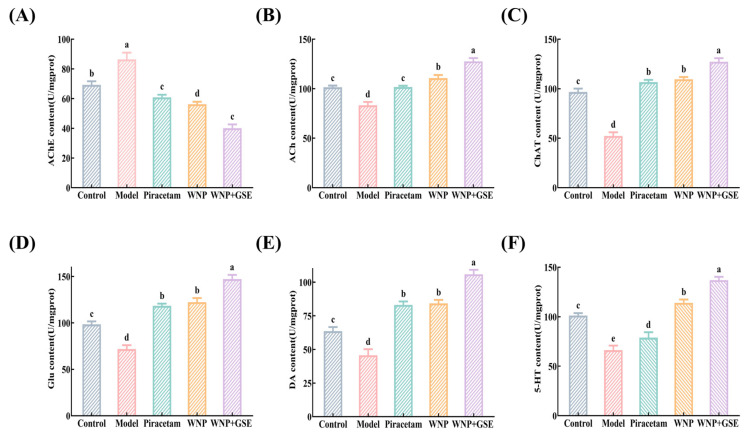
Effect of WNP + GSE on neurotransmitters in serum. (**A**) AChE content in mice serum. (**B**) ACh content in serum. (**C**) Serum ChAT levels in serum. (**D**) Glu levels in mice serum. (**E**) Serum DA content in serum. (**F**) Serum 5-HT content in serum. Data are expressed as means ± standard deviation; all experiments were repeated three times (N = 3). Different letters represent significant differences, *p* < 0.05.

It has been reported that changes in synaptic structure can promote the release of synaptic neurotransmitters. In its early stages, AD is characterized by loss of neurotransmitters and impairment of the cholinergic system, leading to disruption of energy metabolism in the brain and limited neurotransmitter production [33]. The levels of four serum neurotransmitters associated with cognitive function, namely, ACh, Glu, DA, and 5-HT, were monitored in mice. According to the cholinergic hypothesis, the transmission of signals by these neurotransmitters is regulated by AChE, which hydrolyzes excessive ACh in the synaptic gap through nicotinic and muscarinic receptors. The hydrolysis of ACh by AChE disrupts the dynamic balance, resulting in a significant decrease in the number of cholinergic neurons in the basal forebrain and a reduction in ACh levels in presynaptic cholinergic terminals. This imbalance leads to damage or loss of cholinergic neurons in the brain [34]. The results of this work showed that serum AChE levels were higher in the model group (86.37 ± 3.73%) than in the control group (69.25 ± 1.95%) (*p <* 0.05), while the WNP + GSE group (40.05 ± 2.09%) had lower AChE levels than the WNP group (56.19 ± 1.41%) (Figure 5A, *p <* 0.05). Compared to the WNP group (110.68 ± 2.59%), the serum ACh levels were significantly higher in the WNP + GSE group (127.633 ± 4.17%) (Figure 5B; *p* < 0.05), while serum ChAT activity was higher in the WNP + GSE group (127.24 ± 4.50%) than in the WNP group (109.53 ± 1.87%) (Figure 5C; *p <* 0.05). Neurotransmitter ACh is most closely associated with learning and memory and is highly dependent on ChAT for its synthesis. Therefore, AChE, ACh, and ChAT activity are critical for the recovery of cognitive function. Wang et al. [35] demonstrated that the peptide FY (Phe-Tyr) could inhibit AChE activity through Tyr124, Gly122, and Trp86 sites and improve cognitive impairment. Consistently with the above studies, our findings suggest that WNP and WNP + GSE can improve cholinergic dysfunction-induced cognitive impairment, possibly by binding to AChE residues to inhibit its activity and elevate ACh content.

Monoamine neurotransmitters such as 5-HT, DA and Glu may play an equally significant role in influencing cognitive function. Glu, as an excitatory neurotransmitter, can chelate Cu^2+^ to prevent the oxidation of monoamine neurotransmitters and the formation of hydroxyl radicals. It also helps maintain homeostasis and interacts with monoamine neurotransmitters to enhance cognitive function [36]. The results of this work showed that serum Glu levels were significantly elevated in the WNP + GSE group (147.26 ± 3.67%) compared to the WNP group (122.34 ± 3.66%) (Figure 5D; *p <* 0.05). Similarly, serum DA levels were significantly higher in the WNP + GSE group (105.86 ± 2.75%) than in the WNP group (84.23 ± 2.16%) (Figure 5E; *p <* 0.05). Serum 5-HT levels were also found to increase in the WNP + GSE group than in the WNP alone group (Figure 5F; *p <* 0.05). Recinella et al. [37] reported that aqueous extracts of Tanacetum parthenium modulated neuroinflammation by increasing DA release and DA transporter proteins in hypothalamic HypoE22 cells. Wu et al. [38] demonstrated that ginsenoside hydrolysate products increased NA and DA levels in the brain and improved cognitive dysfunction caused by simulated prolonged spaceflight. In conclusion, our results indicate that the combined treatment of WNP + GSE containing various ginsenosides and peptides exhibits synergistic effects. This combination treatment surpasses the effects of WNP alone by modulating neurotransmitter function and restoring the cholinergic system. Ultimately, this intervention leads to improved cognitive performance.

### 3.5. WNP + GSE Stimulated CREB/BDNF/TrkB Protein in the Hippocampus

**Figure 6:**
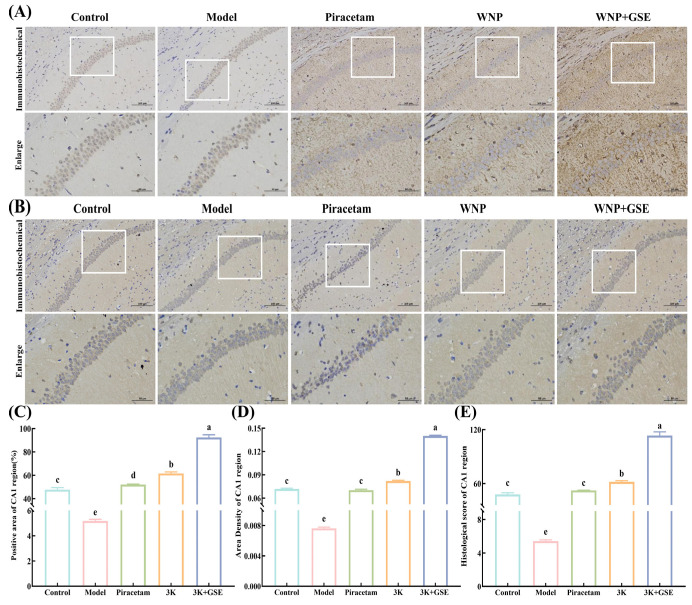

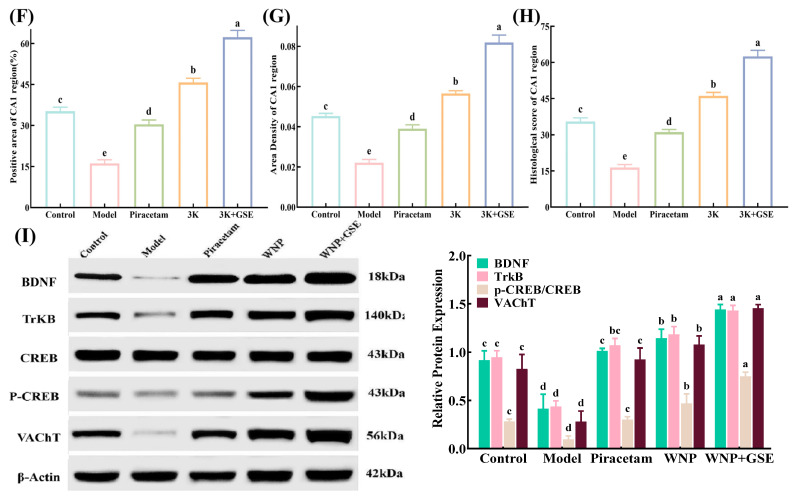
Effect of WNP + GSE on the CREB/BDNF/TrkB signaling pathway. (**A**) Immunohistochemical staining image of BDNF in the hippocampal CA1 region. (**B**) Immunohistochemical staining imaging map of VAChT in the hippocampal CA1 region. (**C**) The ratio of BDNF-positive surface in the hippocampal CA1 region. (**D**) Density of BDNF-positive surface in the hippocampal CA1 region. (**E**) Histochemical score of BDNF in the hippocampal CA1 region. (**F**) The ratio of VAChT-positive surface in the hippocampal CA1 region. (**G**) Density of VAChT-positive surface in the hippocampal CA1 region. (**H**) Histochemical score of VAChT in mouse hippocampal CA1 region. (**I**) Effects of WNP + GSE on the BDNF/TrkB/CREB signaling pathway and VAChT levels. Data are expressed as means ± standard deviation; all experiments were repeated three times; N = 3. Different letters represent significant differences, *p* < 0.05.

In the brain, the enhancement of CREB/BDNF/TrkB signaling is an important factor in increasing neuroplasticity and promoting long term memory. Considerable research has demonstrated low expression of proteins of the CREB/BDNF/TrkB pathway and nerve damage to the hippocampus in rats with memory impairment. To explore whether neurotrophic deletion was involved the memory impairments in the WNP + GSE-treated rats, we measured the expression of CREB/BDNF/TrkB and VAChT. The immunohistochemical image of BDNF in the hippocampal CA1 region of mice in the WNP and WNP + GSE group showed a brownish-yellow and brownish-brown color, respectively, with strong antigen–antibody binding ability (Figure 6A). Quantitative analysis revealed a significant increase in the ratio of positive area, density of BDNF-positive surface, and histochemical scores of the CA1 region in the WNP and WNP + GSE group compared to the model group (Figure 6C–E). Notably, compared to the WNP group (61.60 ± 1.11%), the positive area ratio was higher in the WNP + GSE group (92.42 ± 1.88%) (*p <* 0.05). The trend was also seen in the results of immunohistochemical staining of VAChT in the hippocampal CA1 region (Figure 6B). Similarly, VAChT content in the hippocampal CA1 area of mice was significantly higher in the WNP + GSE group (62.25 ± 2.07%) than the WNP group (45.74 ± 1.25%) (Figure 6F–H; *p <* 0.05). In addition, the WNP + GSE group was found to have higher surface density and histochemical score than the model group (*p <* 0.05).

Western blotting was used to determine the effect of WNP + GSE on the VAChT and BDNF/TrkB/CREB pathway. WNP and WNP + GSE treatment alleviated the low expression of the CREB/BDNF/TrkB pathway induced by SCOP. There was a significant increase in the p-CREB/CREB ratio in the WNP + GSE group (0.75 ± 0.04%) compared to the WNP group (0.47 ± 0.08%) (*p <* 0.05). Levels of TrkB and BDNF were higher in the WNP + GSE group than the WNP group (*p <* 0.05). VAChT protein levels in the model group (0.28 ± 0.008%) were lower than those in the control group (0.83 ± 0.12%) (Figure 6I; *p <* 0.05). After WNP + GSE (1.42 ± 0.26%) intervention, VAChT protein levels were significantly increased compared to the WNP-alone treatment (1.08 ± 0.15%) (*p <* 0.05).

Damage to central cholinergic neurons causes a reduction in the level of the central choline biomarker VAChT, which cannot properly wrap ACh and release it into the synaptic gap [39], thus reducing the concentration of neurotransmitters in the synaptic gap and affecting the secretion and synthesis of BDNF. BDNF is ubiquitously present in the brain and plays a crucial role in regulating neural activity, facilitating local synaptic neurotransmission, and modifying synaptic structure, all of which are closely associated with learning and memory functions. TrkB, on the other hand, serves as a specific receptor for BDNF. The interaction between BDNF and TrkB regulates neuronal survival and differentiation, modulates LTP, and influences synaptic structure and functions. CREB activity is regulated by multiple phosphorylation modifications on it, and CREB phosphorylated at the serine 133 sites continuously induces BDNF expression. BDNF being a direct target of CREB, the binding of activated CREB to BDNF rescues cognitive deficits, so the BDNF/TrkB/CREB signaling pathway is critical for normal learning and memory abilities [40]. Zhang et al. [41] demonstrated that the peptide WCPFSRSF regulates synaptic plasticity and reverses Glu-induced neuronal damage through activation of the BDNF/TrkB/CREB signaling pathway. Lee et al. [42] proved that GSE significantly elevated VAChT to improve cholinergic function and aging-caused cognitive impairment. Wang et al. [28] proved that ginsenoside Rb1 masked miRNA-134 to promote the BDNF pathway and regulate synaptic plasticity in the hippocampal region, as well as the expression of synapse-associated proteins. In summary, WNP + GSE intervention was found superior to WNP alone by promoting BDNF secretion and VAChT expression due to the synergistic effects of peptides and ginsenosides present in GSE, which enhance cholinergic function, increase neurotransmitter content, and activate the BDNF/TrkB/CREB signaling pathway. However, whether WNP + GSE can specifically stimulate the BDNF/TrkB/CREB pathway is still unknown.

### 3.6. Possible Mechanisms of WNP + GSE on Enhancing Learning and Memory Ability

**Figure 7:**
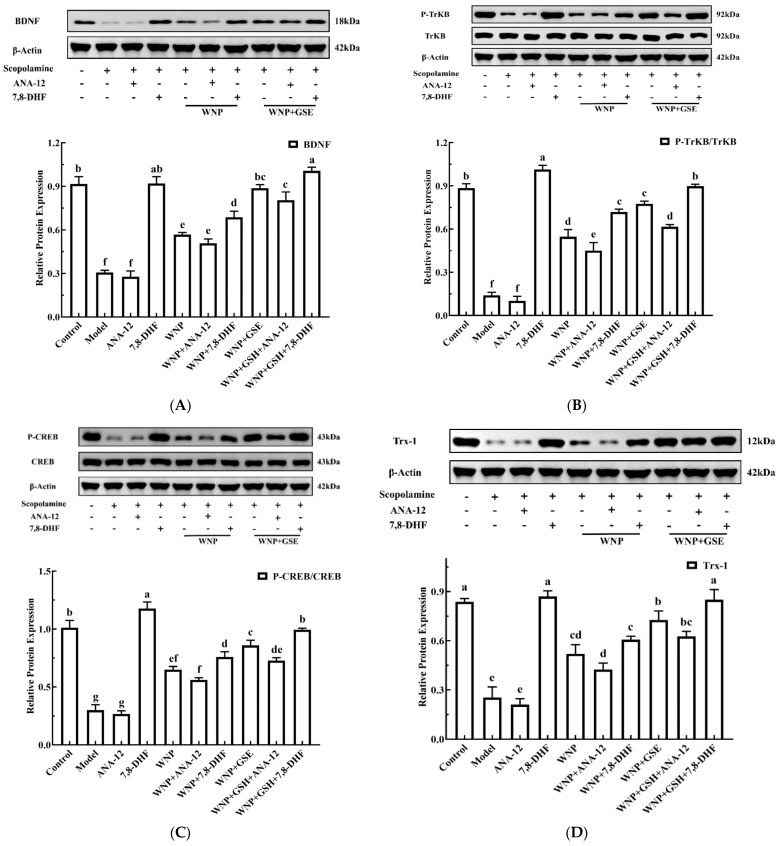
Regulatory mechanisms of WNP + GSE in the BDNF signaling pathway in PC12 cells. (**A**) BDNF levels in PC12 cells. (**B**) P−TrkB/TrkB levels in PC12 cells. (**C**) P−CREB/CREB levels in PC12 cells. (**D**) Trx−1 levels in PC12 cells. Data are expressed as means ± standard deviation; all experiments were repeated three times; N = 3. Different letters represent significant differences, *p* < 0.05.

The previous results showed that WNP + GSE can increase the levels of the neurotransmitter, modulate cholinergic function, and improve cognitive impairment. Next, combination experiments of activators and inhibitors were designed by using a SCOP-induced PC12 cell model to determine if WNP + GSE can specifically stimulate BDNF/TrkB/CREB pathway. As depicted in Figure 6A, consistent with the findings in C57BL/6 mice, the levels of BDNF were significantly diminished in PC12 cells following treatment with SCOP (control group: 0.92 ± 0.04, model group: 0.31 ± 0.01; *p <* 0.05). However, treatment with WNP and WNP + GSE effectively mitigated the downregulation of the CREB/BDNF/TrkB pathway induced by SCOP (*p <* 0.05). Notably, the Trx-1 level was found to be higher in the WNP and WNP + GSE groups than that in the model group (*p* < 0.05). ANA-12 is a potent and highly selective TrkB antagonist, which can cross the blood–brain barrier and exert central TrkB blockade without compromising neuron survival. In PC12 cells treated with ANA-12, the expression of the CREB/BDNF/TrkB/Trx-1 was markedly decreased when compared with the control group and did not show significant differences compared with model groups, but the promoting effects of WNP + GSE were not reversed by blocking the CREB/BDNF/TrkB pathway by ANA-12. For example, BDNF levels were significantly higher in the WNP + GSE group (0.80 ± 0.05) than in the WNP group (0.51 ± 0.02; *p <* 0.05, Figure 7A). In Figure 7B, the p-TrkB/TrkB levels are higher in the WNP + GSE group (0.62 ± 0.01) than in the WNP group (0.45 ± 0.04) after treatment of PC12 cells with ANA-12 (*p <* 0.05). The same trends were also observed for P-CREB/CREB levels and Trx-1 expression (Figure 7C,D). Meanwhile, the agonist effect of 7,8-DHF confirms that the TrkB receptor is the specific target for 7,8-DHF. These findings suggested that in vivo, WNP + GSE and 7,8-DHF did not exhibit synergistic or additive effects (Figure 7A–D). However, the potency of WNP + GSE was greater than WNP alone. For instance, in PC12 cells treated with 7,8-DHF, the WNP + GSE group showed a higher BDNF protein level than the WNP group (1.01 ± 0.02 vs. 0.69 ± 0.03, *p <* 0.05). The WNP + GSE group had higher P-TrkB/TrkB levels (0.90 ± 0.01) than the WNP group (0.72 ± 0.02) (*p <* 0.05). The same trends were also observed for P-CREB/CREB levels and Trx-1 expression (Figure 7C,D).

Trx-1, one of the major neurotrophic factors in cholinergic neurons, promotes neuronal development. It acts as an antioxidant regulatory protein and reduces Cys-32 and Cys-35 sites on cysteine residues in cells, protects neurons from apoptosis, and delays AD development [43]. Zhang et al. [44] showed that Trx-1 improved cognitive impairment by restoring the expression of neurotransmitter receptors and increasing CREB phosphorylation. Wang et al. [45] reported that ginsenoside R1 attenuated mitochondria-mediated apoptosis and thus prevented acrylamide-induced neurotoxicity by increasing Trx-1 levels. The findings from this study suggest that the combination of WNP + GSE has the potential to promote the expression of BDNF and its binding to the high-affinity receptor TrkB. This activation is primarily achieved through phosphorylation at the Y515 and Y816 sites, leading to subsequent activation of the Ser133 site on CREB. Ultimately, this phosphorylation cascade leads to continuous induction of BDNF expression and restoration of cholinergic function. Increased BDNF and CREB levels induce Trx-1 expression and enhance synaptic plasticity and neuronal proliferation. However, these kinds of effects were not inhibited or boosted by ANA-12 or 7,8-DHF, which indicates that other mechanisms of memory improvement of WNP + GSE must exist. WNP + GSE boosted memory via multiple targets and multiple pathways.

## 4. Conclusions

The present study indicated that there is a synergistic interaction between WNP and GSE and that WNP + GSE can be used as an effective memory improvement supplement. The main mechanism through which WNP + GSE enhances memory function is by promoting neuronal integrity and synaptic plasticity. The synergistic effects of WNP + GSE on memory improvement can be attributed to several factors. These include the increased accumulation of neurotransmitters, such as AChE, ACh, ChAT, Glu, DA, and 5-HT, activation of the BDNF/TrkB/CREB pathway, regulation of cholinergic function, and elevation of Trx-1 protein levels. Notably, WNP + GSE boosted memory via multiple pathways, not only the BDNF/TrkB/CREB target. WNP + GSE had the synergistic ability to ameliorate the learning and memory impairment induced by SCOP, which inspired the further application of WNP + GSE in functional food.

## Data Availability

Data is contained within the article.

## Abbreviations

Acetylcholine (ACh); acetylcholinesterase (ache); Alzheimer’s disease (AD); brain-derived neurotrophic factor (BDNF); choline acetyltransferase (chat); cAMP response element-binding protein (CREB); dopamine (DA); glutamate (Glu); glutathione peroxidase (GSH-Px); ginseng extract (GSE); hematoxylin and eosin (HE); long-term potentiation (LTP); Morris water maze (MWM); phosphorylated cAMP response element-binding protein (p-CREB); phosphorylated tyrosine kinase B (p-TrkB); scopolamine (SCOP); tyrosine kinase B (TrkB); thioredoxin 1 (Trx-1); transmission electron microscopy (TEM); vesicular acetylcholine transporter protein (vacht); walnut peptide (WNP); 5-hydroxytryptamine (5-HT); 7,8-dihydroxyflavone (7,8-DHF).
