# Correction: Fu et al. Synergistic Effect of Combined Walnut Peptide and Ginseng Extracts on Memory Improvement in C57BL/6 Mice and Potential Mechanism Exploration. *Foods* 2023, *12*, 2329

**DOI:** 10.3390/foods14040672

**Published:** 2025-02-17

**Authors:** Junxi Fu, Wentian Song, Xiaobing Song, Li Fang, Xiyan Wang, Yue Leng, Ji Wang, Chunlei Liu, Weihong Min

**Affiliations:** 1 College of Food Science and Engineering, Jilin Agricultural University, Changchun 130118, China; 2 National Engineering Laboratory of Wheat and Corn Deep Processing, Changchun 130118, China; 3 Zhongke Special Food Institute, Changchun 130022, China

The authors would like to make the following correction to a published paper [1]. In the original publication, Figure 1 included a duplicated image. The correct Figure 1B,C and the corresponding parts of the caption are shown below. The authors state that the scientific conclusions are unaffected. This correction was approved by the Academic Editor. The original publication has also been updated.

## Figures and Tables

**Figure 1:**
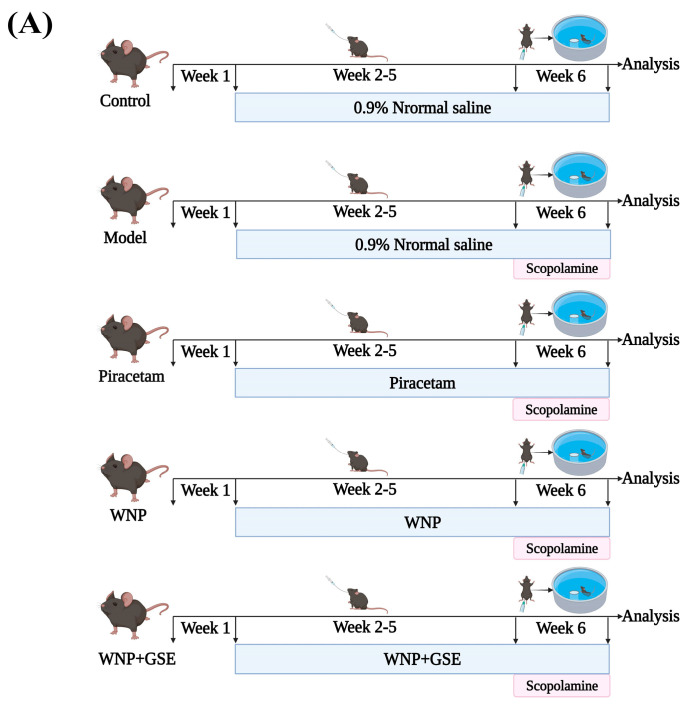

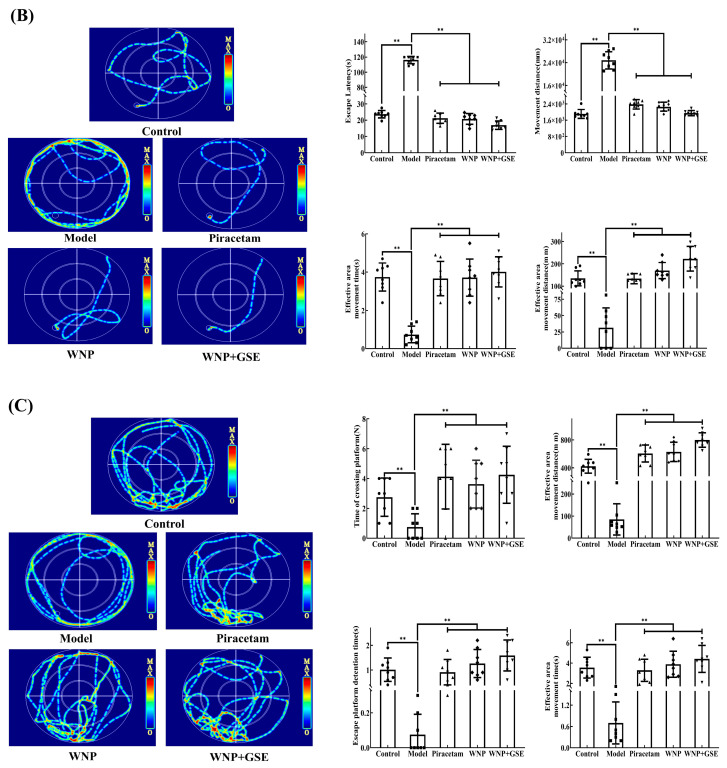
Effects of WNP + GSE on learning and memory deficits of SCOP–induced mice (**A**) Flow diagram of experimental tests. (**B**) Representative image of movement trajectory of mice in the positioning navigation test, including mice escape latency, total movement distance, effective movement time, and effective movement distance. (**C**) Representative image of movement trajectory of mice in the spatial exploration test, including the number of times mice crossed the original platform, effective movement distance, escape residence, and effective movement time. N = 8 mice/group. The data are expressed as mean ± standard deviation. All of the experiments were repeated three times. ** indicate significant difference between groups at *p* < 0.01.
